# Implementation Science Workshop: Engaging Patients in Team-Based Practice Redesign — Critical Reflections on Program Design

**DOI:** 10.1007/s11606-016-3656-8

**Published:** 2016-03-14

**Authors:** Sarah Davis, Stephanie Berkson, Martha E. Gaines, Pratik Prajapati, William Schwab, Nancy Pandhi, Susan Edgman-Levitan

**Affiliations:** Center for Patient Partnerships, University of Wisconsin, 975 Bascom Mall - Suite 4311, Madison, WI 53706 USA; University of Wisconsin Law School, Madison, WI USA; University of Wisconsin Medical Foundation, Madison, WI USA; Department of Family Medicine and Community Health, University of Wisconsin School of Medicine and Public Health, Madison, WI USA; Division of General Internal Medicine, Massachusetts General Hospital, Boston, MA USA; John D. Stoeckle Center for Primary Care Innovation, Massachusetts General Hospital, Boston, MA USA

## CASE

### Introduction

Redesigning the US healthcare system to support patient-centered primary care has become a national priority to improve quality.^1^–^3^ Models for primary care redesign both promote the shift towards team-based patient-centered care^4^–^6^ and set an expectation for front-line teams to improve and standardize processes for improved patient experience outcomes.^7^–^10^ Engaging patients in these quality improvement (QI) efforts is increasingly viewed as essential.^11^–^16^ Despite its value, there are few evidence-based methods or practical toolkits for engaging patients in QI, and fewer still that focus on clinical care teams.^17^

This article introduces a patient engagement program for team-based practice redesign efforts. UW Health, a large Midwestern academic medical center, underwent a comprehensive primary care redesign which included strategies at the system, clinic, and care team levels. At the care team level, the redesign initiative adopted a microsystem approach,^18^–^21^ using trained coaches to support team and QI skill development across five team cohorts over 4 years. It turned to consultants at the Center for Patient Partnerships (CPP), a patient advocacy center housed at the University of Wisconsin-Madison, to collaboratively develop a program which engaged volunteer patients as part of team QI efforts.

### Setting and Participants

UW Health has 279 primary care physicians (family physicians, general internists, general pediatricians) across 40 primary care clinics located primarily in Madison and surrounding Dane County, Wisconsin. Primary care is responsible for ~279,000 medically-homed patients. Patients are considered medically-homed if they had an identified primary care provider and a telephone contact or clinic visit in the past three years. Forty-nine microsystem teams from 26 of these clinics volunteered to participate in the initiative. Groups comprising 9 to 11 teams received training together as a cohort. Participating clinics were primarily located in urban settings; however, the sociodemographic characteristics of the communities served varied in terms of race, language spoken, and insurance coverage. A typical microsystem team consisted of a physician, physician assistant or nurse practitioner, nurse, medical assistant, and receptionist. Prior to the initiative, teams had not engaged in team-sustaining activities on a regular basis.

### Program Description

The overarching goal of the program was to lead the culture change necessary to inspire and normalize patient engagement activities. Consultants from CPP designed a customizable training program and accompanying toolkits with initiative-level, coach-level, and team-level interventions. Program materials incorporated items such as recruitment letters, patient job descriptions, and surveys created by the teams (*see*Appendix). Sample toolkits are available on the University of Wisconsin’s Health Innovation Program website, HIPxChange (www.hipxchange.org).

**Figure 1:**
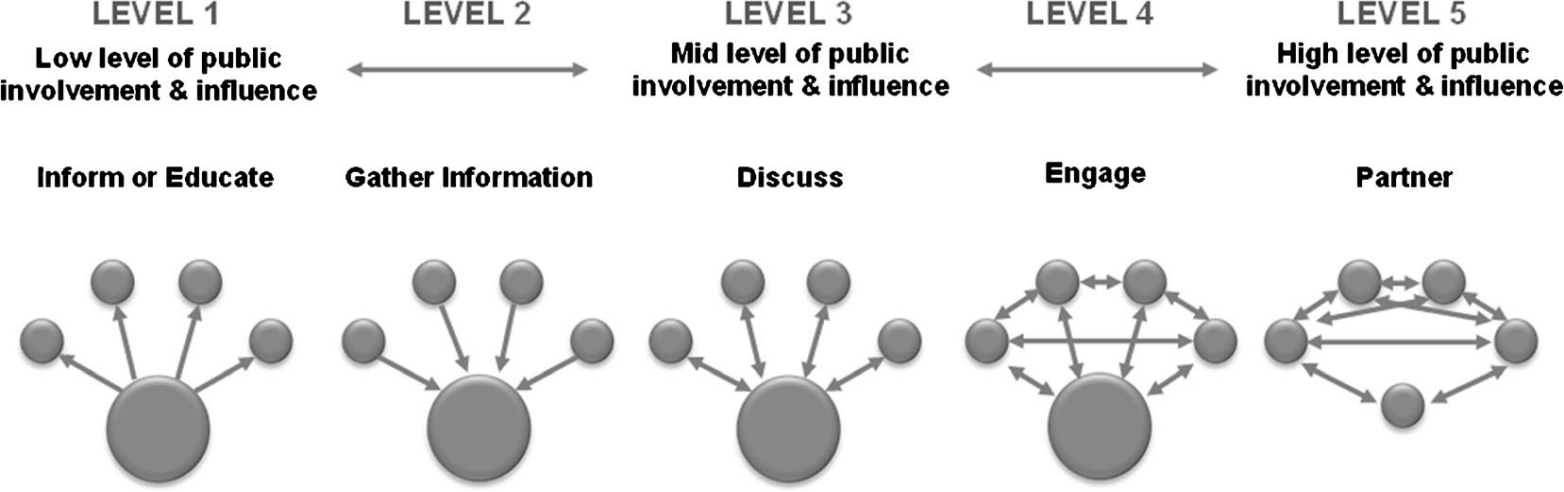
Health Canada Public Involvement Continuum. The program adapted this framework by replacing “public” with “patient.” It visually displays progressive levels of patient engagement along a spectrum including the directional nature of activities and corresponding patient influence

The program offered a framework adapted from the Health Canada Public Involvement Continuum, by replacing “public” with “patient.” It visually displays progressive levels of patient engagement along the spectrum of inform, gather, discuss, engage, and partner (*see* Fig. 1).^22^ Through training activities and the toolkit, teams were encouraged to mix and match engagement activities to maximize input from patients, and were taught that all engagement is valuable. While the program communicated that higher levels of engagement would yield richer input, it also reinforced that all engagement activities offered the patient perspective; it was better to engage patients at a lower level than not at all. For example, teams “gathered” data through surveys and cycle times, engaged in “discussions” with patients in one-time focus groups, “involved” patients through ongoing advisory panels, and “partnered” with patients by inviting them to join their QI team. Engagement efforts had specific QI goals in mind: one team solicited in-person feedback regarding the timing of delivering pediatric immunizations during an office visit, while another team held a focus group to help their clinic redesign the waiting room. Additional examples are provided in Table 1.

**Table 1:** Patient Engagement Activities by Participating Quality Improvement Teams Across Five Cohorts (*N* = 49)

Highest patient engagement level achieved*	Number of teams	% of teams	Activities	Patient engagement contributions
None	5	10 %	N/A	N/A
Level 1: Inform/ Educate	0	0 %	Visibility walls, patient education materials	Making posters and brochures about online patient portal visible and available, team considers patient perspective in how to communicate changes.
Level 2: Gather	20	41 %	Surveys, cycle times, penless surveys, phone surveys, interviews, in-person feedback, paper surveys	Cycle time data collected by patients led team to decrease wait time in the exam room. Patient feedback about receiving immunizations prior to physician visit (90 % supported) led this process change to be the standard.
Level 3: Discuss	18	37 %	Focus groups, phone conversations, team meetings, phone interviews, paper surveys, interviews in clinic, email feedback, phone surveys, asking for patient feedback on group visits	Phone discussions with patients led to highlighting physician instructions on the After Visit Summary.
Level 4: Involve	4	8 %	Advisory panels, patient panel, focus group	Focus group helped develop a communication to parents to help prepare for adolescent appointments. Focus group discussion led to change in waiting room layout to make it more welcoming and conversational.
Level 5: Partner	2	4 %	Team member	Patient participating in team meetings changed the conversation and helped to shift the culture (e.g., patients considered partners in care, not external customers)

*All teams were trained to “mix and match” engagement activities on various levels in order to maximize input from patients, and were trained that all engagement is valuable. When teams utilized more than one method, the highest level was recorded. Beginning with cohort 2, teams were required to engage patients at least at the “gather” level.

Training and materials were developed and coordinated by consultants for three distinct levels: program, coach, and team. Training explained the why and how of patient engagement, including assessing team readiness, selecting the best engagement methods for the QI project, recruiting patients, and purposefully selecting patients for diverse input.

Activities were incorporated into the initiative’s large learning sessions (1–2 times per 6–9-month cycle; *see*Appendix for further information), sessions for coaches (3 per cycle), and team-based meetings (varied). Team training guided teams in the appropriate mix of engagement methods and patient selection for their specific QI effort. For example, a team moving to a new clinic was encouraged to draw from a cross section of their patient panel to maximize broad input, while the team looking to change their immunization protocol only surveyed parents at visits where immunizations were scheduled. The physician from the relocating clinic reported: “We asked all providers to give us names of patients they thought would be interested in volunteering, considering diversity in sex, age, etc. We reviewed the list for diversity and then called ~40 patients and sent information about the opportunity, which resulted in ten patients participating.”

When teams struggled with engaging patients, consultants reached out to coaches to offer additional support and offered to meet with teams. Consultants simultaneously worked on clarifying organizational policies to facilitate engagement, including the role of patient partners within the organization, the impact of the Health Insurance Portability and Accountability Act (HIPAA), and reimbursement for nominal expenses. Consistent with the overall initiative, the program used a train-the-trainer approach, in which consultants trained coaches, who then primarily trained the teams. Consultants were actively involved for the first and second cohorts, transferring oversight to coaches midway through cohort 3.

Program implementation was modified in real time based upon feedback from evaluations and participant discussions. This program enhances existing frameworks by including engagement at the practice/microsystem/team location, specifically for QI efforts.^23^,^24^ The toolkits can be implemented in other settings with minimal customization compared to other available resources, as they are prepackaged for end users.^17^

### Program Evaluation

#### Methods

Program evaluation was part of an ongoing evaluation of the initiative, which was designed using an outcomes-oriented approach where objectives and corresponding outcome measures were established and then tracked.^25^ No comparison group was established for the intervention, as it was a naturally occurring experiment in operating clinics. This project was determined to be exempt from institutional review board review.

Program outcome measures were penetration, acceptability, adoption, cost, and appropriateness/feasibility. Five sources of data were collected to assess these measures, as follows: *penetration* data were obtained from (1) the tracking log of teams that had engaged patients and the types and levels of activities and (2) responses on patient experience of care surveys; *acceptability* data came from (3) closed- and open-ended survey responses and (4) consultant reports; *adoption* was assessed using (1) the tracking log and (4) consultant reports; *costs* were calculated through reviewing (5) consultant invoices and additional nominal internal cost estimates related to the program (e.g., food and transportation that supported patient engagement); *appropriateness/feasibility* was assessed through analysis of (3) open-ended survey responses and (4) consultant reports.

Data collection and analysis for the above five sources proceeded as follows. First, the redesign initiative tracked the percentage of teams trained in the intervention, allowing for calculation of penetration. It provided a log of the types and levels of activities that participating teams conducted, which was used to determine adoption. Penetration was assessed through responses to a standardized mail survey of 45 questions sent by Avatar International to a randomly selected group of patients seen in clinic between October 2010 and May 2012. Avatar International provides the survey infrastructure for patient satisfaction reporting at the organization. Questions covered domains such as availability, timeliness, physician care, and office staff care. Responses were divided into two groups according to whether a primary care physician was a program participant. The percentage of responses indicating “strongly agree” for each question was then compared using a *t* test. Responses were considered significant at *p* < 0.05.

Next, an anonymous email survey was distributed at baseline and at 6 and 12 months to the 134 team members (not including patient members) who completed the program during the first four cohorts. Three closed-ended queries specific to patient engagement acceptability were asked (*see* Table 2). Response categories were based on a five-point Likert scale (strongly disagree, disagree, neutral, agree, and strongly agree). Differences between responses at baseline and 6 months and between baseline and 12 months were tested with the Mann–Whitney *U* test. Differences were considered significant at *p* < 0.05. Additionally, non-patient team members from the first trained cohort were asked to “list two things you learned about engaging patients during the past 6 months” and “what, if anything, needs to happen to enable your team to engage patients?” These open-ended responses were analyzed independently by three authors using content analysis, and then discussed to reconcile differences and identify overall summary themes for acceptability and appropriateness/feasibility.

**Table 2:** Patient Engagement Survey Responses Across Four Completed Cohorts

Survey Question	Timing	Responses					*p* value (pre/post)
		Strongly disagree	Disagree	Neutral	Agree	Strongly agree	
I believe that patients bring a perspective to a project that no one else can provide.	Baseline *N* = 134	0 %	3 %	12 %	57 %	28 %	
	6 months *N* = 103	0 %	2 %	4 %	54 %	40 %	0.01
	12 months *N* = 98	0 %	0 %	11 %	48 %	41 %	0.04
I am confident of my ability to engage patients in microsystem improvement work.	Baseline *N* = 134	1 %	4 %	30 %	52 %	13 %	
	6 months *N* = 103	0 %	2 %	20 %	60 %	17 %	0.03
	12 months *N* = 98	0 %	7 %	18 %	49 %	25 %	0.03
I am interested in engaging patients in microsystem improvement work.	Baseline *N* = 133	1 %	4 %	30 %	49 %	16 %	
	6 months *N* = 103	0 %	0 %	15 %	56 %	29 %	<0.0001

Next, content analysis was performed on the nine monthly and two final cohort progress reports written by consultants. These reports included a status update, summary of deliverables, notes on teams’ progress, and process reflections. This analysis added detail to the acceptability, adoption, and appropriateness/feasibility measures.

The final data source was an internal summation of program expenditures provided by the initiative. This was divided by the total number of teams trained to determine cost per team.

#### Results

##### Penetration

The initiative began with nine teams in the first cohort, and spread to 49 teams by cohort 5. The 49 trained teams were spread across 26 clinics (approximately two-thirds of UW Health’s primary care clinics), with the 44 teams who engaged patients representing 60 % of clinics. In addition, the project expanded to assist with patient engagement through a newly formed Diabetes Patient Advisory Council, a patient representative joining the UW Health Improvement System Advisory Council, and two clinic-level patient advisory panels.

Participating teams had significantly higher ratings than non-participating teams on 71 % of patient experience questions (32/45). Differences were as high as four percentage points (data not shown). Questions for which there was no difference generally dealt with the overall facility (e.g., waiting room comfort). When asked about agreement with the statement “My doctor’s office has a very high quality of care,” 89 % of participating team patients strongly agreed, compared to 87 % of non-participating team patients (*p* < 0.05).

##### Acceptability

Across four cohorts, survey response rates varied from 85 to 99 % at baseline, 59 to 79 % at 6 months, and 52 to 88 % at 12 months. Over time, survey response results revealed that a significantly increased percentage of respondents believed (agreed or strongly agreed) that patients brought a vital perspective to a project, were confident of their ability to engage patients in microsystem improvement work, and were interested in doing so (Table 2). Content analysis of consultant reports revealed that acceptability varied by individual, audience, and time. For example, in January 2011, consultants reported that after the official end of cohort 1, during which patient engagement was not an expected deliverable, all but two teams had “stalled” in engaging patients. Additionally, in their June 2011 report, they noted: “A few [cohort 2] coaches still seem resigned that their teams ‘don't see a role for patients’ in their redesign work…”

Themes from open-ended team survey questions revealed that participants learned that incorporating the patient perspective is valuable. They also learned that patients want to engage with teams and appreciate the opportunity.

##### Adoption

Table 1 shows engagement activities and resulting contributions according to highest level of patient engagement. Of the teams included in this study, 92 % engaged patients in QI activities (cohort 1 [9 teams] = 70 %, cohort 2 [9] and 3 [10] =100 %, cohort 4 [11] = 91 %, cohort 5 [10] =100 %). Content analysis of consultant reports revealed that having a strong champion, usually either the coach or physician lead, and/or motivation to improve a situation (e.g., moving clinic, inefficiencies in daily schedule) was correlated with greater levels of engagement. Teams that did not engage patients cited both competing demands on their time, such as electronic medical records implementation, and a lack of valuing the engagement of patients.

##### Cost

The program built on existing improvement education infrastructure, utilizing QI leadership and improvement coaches. For the 49 teams, additional incremental costs were ~ $1,200/team. Initial expenses were highest to invest in training and tool development, and decreased significantly by cohort 3. These capabilities are now part of the internal QI infrastructure, and additional incremental costs are expected to be minimal.

##### Appropriateness and Feasibility

From the open-ended response analysis, specific preparation, planning, and skill cultivation were necessary for teams to engage patients. Necessary conditions included financial support and protected time to engage in this work, agreement about the importance of patient engagement, and a collective commitment to the work involved. Consultants’ reports revealed the need for protected time. Engaging patients was perceived as an appropriate component of team QI work when integrated into existing work and engagement methods matched the relevant improvement. Clarifying organizational policies relevant to patient engagement was also crucial for feasibility. Consultant’s July 2010 report stated: “Coaches expressed a need to address real and perceived organizational barriers to ‘clear the way’ for active patient engagement.”

### Challenges and Future Plans

The program was designed to adapt to provider and team variation in terms of preparedness (regarding ability, interest, time, and readiness) to engage patients in care redesign.^26^,^27^ Initial participants expressed skepticism about team ability to engage patients and organizational commitment. Significant practical limitations were offset to the extent possible by using coaches to lead the efforts and involvement of all team members (e.g., patient outreach by receptionists). Demonstrating the value of engaging patients in improvement work and gaining experience or skills helped to overcome lack of support/enthusiasm for some teams, but not others. Over time, evaluation results demonstrated a change in primary care team attitudes about patient engagement. Teams who engaged patients changed processes, communications, and physical layouts as a result. For example, one team moved immunizations earlier in the visit, and another improved instructions on self-rooming in a new clinic (*see* Table 1).

Teams who did not engage patients cited both competing priorities and lack of belief in the value of engaging patients as impediments to participation. We believe the program could be improved by proactive organizational policy development that prepares the organization for a patient engagement program, allocates resources to participating teams, requires deeper integration with other QI activities, and creates expectations for more long-term engagement.^28^

#### Limitations and Need for Additional Research

Our findings and recommendations are subject to several limitations. First, our program was implemented at a large academic health system, and thus may lack generalizability. Second, the program was designed with QI rather than research in mind, so standardized data were not gathered from the overstretched teams on process measures, such as patient recruitment efforts. Next, survey response rates declined over time. While typical of surveys in busy clinical environments,^29^,^30^ there is a risk that we overrepresented perspectives of team members who were satisfied with their training and experiences. Fourth, the difference in patient experience data scores may be due to selection bias, as those who chose to participate in the program were physicians with higher baseline patient ratings.

Lastly, a significant limitation is that our program evaluation did not collect data from patients engaged by teams. Future research would be greatly enhanced by incorporating the patient perspectives on engagement, and particularly exploring patient-level and coach-level factors that lead to success. In addition, future research is needed to explore the relationship between patient engagement and primary care outcome priorities, including care experience, clinical outcomes, efficiency and access, and staff and patient satisfaction.

## TEACHING COMMENTARY

By Susan Edgman-Levitan, PA

If you were building a house, you would be intimately involved with the architect and the contractor, reviewing all plans to ensure that the design and decor worked for your family. You would bring the knowledge of your family’s needs and preferences, and they would bring the technical skills to build a functional, well-designed home. Co-designing the house through a successful partnership surely increases the likelihood of a positive outcome. How often, however, does this partnership happen in healthcare, when we design new programs, processes, or educational materials to improve the quality of health for the people we serve? Typically, the most important “experts”—ordinary people managing their health—are left out of the discussion and treated as objects *of* care, rather than partners *in* care.

Clinicians often assume that they understand the experience of illness. But knowledge about physiology or diagnosis and treatment are not the same as understanding how it feels to be sick or to manage a chronic health problem. Clinicians rarely experience the frustrations and challenges of getting care until they or a family member needs care. Patients bring the “lived” experience, as well as knowledge about how well the healthcare system functions to meet their needs. When clinicians and managers partner with patients to co-design care, they frequently realize that patients know more about their operations and clinical practice than they do.

"Engaging Patients in Team-Based Redesign" by Davis et al. describes a comprehensive patient engagement program conducted in primary care practices affiliated with UW Health in Wisconsin. The research team and the Center for Patient Partnerships recruited patient partners to work on practice redesign quality improvement projects. Practices employed a framework of different levels of patient engagement and were encouraged to use a combination of methods to maximize patient involvement in care redesign. Over time, the evaluation demonstrated positive changes in staff attitudes about the value of partnering with patients. Participating teams had significantly higher ratings on patient experience measures compared to non-participating teams.

The definition of “patient engagement” depends on the stakeholder. However, the ideas of partnership, communication, information exchange, and respect are common to most. In decades past, patients were labeled “difficult” if they were active participants in their care; today we know that optimal care *depends* on the wisdom and experience of patients and families. “Patient-centered care” was coined by the Picker/Commonwealth research team at Beth Israel Deaconess Medical Center in 1988, and then integrated into the quality improvement universe by the Picker Institute. The Picker Institute definition, “health care that establishes a ***partnership among practitioners, patients, and their families***…to ensure that decisions ***respect patients’ wants, needs, and preferences,*** and that patients have the ***education and support*** they need to ***make decisions and participate*** in their own care,”^31^ was included in the 2001 *Crossing the Quality Chasm* study^32^ and became one of the six aims for improving the overall quality of the US health care system.

Patient engagement is multi-dimensional: it can be very personal, such as a shared decision between a patient and healthcare professional, or it can be a systematic public event, such as a campaign to improve health literacy. It can also improve provider performance, such as when patient and family advisors and healthcare professionals redesign healthcare services together, or enhance patient behavior through self-management programs for chronic disease. Whatever form it takes, engagement changes the focus from “taking action to improve health and healthcare *for* the people, to taking action *with* the people”—a simple yet radical notion.

This study shares a useful framework developed by the Health Canada Public Involvement Continuum for matching the level of patient involvement to the task at hand. The framework describes progressive levels of involvement moving from **inform** through **gather**, **discuss**, **engage**, and **partner**. This approach allows clinicians to use “small tests of change” to experience why partnering with patients is helpful and to build local “buzz” that helps overcome the concerns of critics. It also helps practices conserve resources. Patients can review and comment on patient education materials on their own, without face-to-face time with practice staff. Improving a practice phone system or a patient portal may require only a focus group. On the other hand, redesigning the physical environment or participating in a root-cause analysis usually requires formal advisory councils with consistent participants. Practices can apply the framework strategically and should be encouraged to use all of the methods, rather than viewing the ultimate goal of **partner** as the “Holy Grail.”

For decades, the Institute for Patient and Family Centered Care has encouraged these concepts through the promotion of patient/family partnerships, but adoption has been slow. Myths abound regarding the value of and barriers to partnerships. These include fear about “showing our dirty laundry,” concern about inappropriate expectations, worry that clinicians and staff will be subjected to anger or criticisms, and assumptions that patients will have nothing to add. Patients and families fear their recommendations will be ignored or that they will be intimidated by clinicians speaking in jargon. When patients and the clinicians with whom they partner are trained using the methods described in the Davis paper, these experiences rarely occur.

The study team used thoughtful implementation strategies to improve the likelihood of success. Because partnering with patients was a new concept for everyone, training and orientation programs were critical. Training for three distinct audiences—practice leaders, practice coaches, and practice teams—was staged over time to introduce and reinforce the new competencies, and well-designed educational materials were essential. Sessions addressed important concerns including how to design orientations for patients and practice teams that focus on topics such as timing of meetings, creating agendas, HIPAA information, eliminating the use of jargon, and reimbursement for patient partners. Implementation across 60 % of their practices is indicative of the value of their study design, as it often takes organizations much longer to garner support for such activities.

The team’s evaluation included many critical factors, including penetration of patient involvement, adoption of patient recommendations, costs, and feasibility. Some important factors, however, were not addressed. The evaluation focused only on the costs of involving patient partners, and not on the savings to practices resulting from patient recommendations. The “hidden impact” of failure to involve patients in care redesign is that initiatives are often underutilized or do not achieve their goals. Sadly, this can waste money and reinforce the perception that patients are not interested in programs and services—when it actually reflects “design failures,” not “end-user failures.”^33^

Failure to interview patient partners about their experiences is also a lost opportunity. These interviews help identify how patient partnership efforts can be improved, the types of support patients and staff need in order to be successful, and other barriers that can be reduced. Research is also needed to determine the most effective engagement strategies for different settings in the healthcare continuum and across different conditions.

Strategies to help overcome resistance to working with patient partners include:

- Starting small, with simple activities such as reviewing patient education materials or a practice website
- Asking clinicians and staff to suggest people who can provide insight and deliver constructive feedback
- Inviting people whose experience of illness is specific to the task and who are available for the task
- Providing training for everyone involved
- Acting on recommendations and evaluating results.

As physicians and other health care clinicians become more “at risk” for the outcomes of their patients, patient engagement at all levels of our health care system will become critical. Many clinicians still employ “magical thinking”—assuming that all of their recommendations are followed by their patients without question—and then become frustrated and burned out because they do not understand why their care is ineffective. Patients have important insights and wisdom that we cannot afford to ignore. Primary care clinicians are deeply committed to the long-term relationships they develop with their patients, and to their role as a trusted healer. These relationships will only become deeper and more effective when we overcome our perceptions of patients and families as passive sources of data rather than active partners in care. When patients and staff come together in these shared co-design efforts, meaningful and sustainable improvements can be made.

In 1988, Harvey Picker, former CEO of Picker X-Ray, supported the Picker Institute’s work because he felt that the healthcare system viewed patients as “imbeciles or inventory.” Fortunately, we have made much progress in changing these attitudes, but we still have a ways to go. I look forward to a day when the clinical paradigm moves from only “What is the matter?” to “What matters to you?”.34 Partnering with patients and their families will take us there, and we will all benefit.

## Electronic supplementary material

Below is the link to the electronic supplementary material.

ESM 1 (DOCX 24 kb)
