# Supplementary material for: Implementation Science Workshop: Engaging Patients in Team-Based Practice Redesign — Critical Reflections on Program Design
Source: J Gen Intern Med. 2016 Mar 14;31(6):688–95. doi: 10.1007/s11606-016-3656-8 (PMC4870427; doi:10.1007/s11606-016-3656-8)
Supplement: Supplementary file 1 — (DOCX 24 kb) [file 11606_2016_3656_MOESM1_ESM.docx]

**APPENDIX**

Co-created program materials for care team-level patient engagement program were designed to be easy to use and practical. They were inspired by existing organizational and system level materials.^1-7^

Trainings included large group sessions, coaches’ sessions, and team-based meetings. Large session activities included panels with patient partners – patients and family members who volunteered to participate in the program – and the providers who engaged them, and small table activities guided by toolkits. Coaches’ sessions included role-playing team meeting discussions about resistance to patient engagement, watching success story video vignettes, and sharing early engagement successes. Team sessions included consultants helping teams select the best engagement method to match their quality improvement objective(s), writing job descriptions for engaged patients, identify appropriate patients to engage, and talking through perceived barriers to engagement to help the teams surmount them.

To guide selection of patient partners, training materials emphasized traits to consider (e.g. challenging healthcare experience, valuable personal experience (cultural background, multiple children), and/or useful professional background) and offered a process under which Patient Partners and the team reach a “mutually beneficial match.” Teams were told that one engagement activity can never be representative of all patients, and were encouraged to utilize various engagement methods (“mixing and matching”) to seek as much input as possible.

Program materials included four separate booklets: (1) “How To” Toolkits; (2) *Patient Partner Welcome Packet template* for patients; (3) *Patient Partner QI Packet: for the Truly “QI” Curious* for patients; and (4) an internal guide including frequently asked questions and relevant policies, *Engaging Patient Partners for Better Care: Getting Started & Vital Resources* (see Table A).

***“How To” Toolkits***

Toolkits were designed with two levels of detail – a more detailed version for coaches, and a more streamlined one for teams. Organized to offer guidance and questions to consider, toolkits present a 6-step “How to” guide with spaces to make notes (Table B). Toolkits were designed to be adaptable, for other initiatives. Acknowledging the newness of engaging patients as a cultivatable skill that benefits from practice and guidance was a critical programmatic element. Introductory text highlights the documented value of patient engagement – that it has improved quality and efficiencies of health systems, increased patient and staff satisfaction, and enhanced mission focus^8^ – and also invites participants to appreciate the intrinsic value of asking patients for their input. Physical copies of toolkits were provided at trainings, and electronic versions were also available through the organization’s intranet.

***Patient Partner Welcome Packet***

The Patient Partner Welcome Packet introduces patients to the philosophy behind engaging patients, the value of contributing to practice redesign efforts, and the specific initiative. It also includes a confidentiality agreement and provides a sample quality improvement (QI) process (from problem assessment to pilot testing). In addition, it offers tips for successful service, such as to generalize from personal health care experiences and appreciate providers’ perspectives.

***The Patient Partner QI Packet: For the Truly “QI” Curious***

This additional patient packet provides even more detail about QI tools and methods for patient partner team members. It includes explanations for teams regarding industrial engineering tools such as the Plan-Do-Check-Act cycle, process mapping, and fishbone diagramming.

***Engaging Patient Partners for Better Care: Getting Started & Vital Resources Guide***

Addressing organizational barriers – real and perceived – and clarifying policies was essential to fostering a culture welcoming to patient engagement. This crucial internal guide was created to: distinguish “patient partners” from “volunteers” for purposes of organizational policy; explain the relationship between Health Insurance Portability and Accountability Act (HIPAA) compliance and patient engagement; address policies and procedures regarding the nominal costs of engagement; introduce internal resources to support engagement (e.g., toolkits, interpreter services, focus group expertise); cover opportunities to celebrate successes through publicity efforts and necessary authorizations; and address confidentiality concerns (and provide a confidentiality agreement).

Sample toolkits and resources are available on the University of Wisconsin’s Health Innovation Program’s dissemination website, HIPxChange (www.hipxchange.org/PatientEngagement).

**APPENDIX REFERENCES**

1. Aurora Health Care, Leonhardt KK, Bonin D, Pagel P. Guide for Developing a Community-Based Patient Safety Advisory Council. Rockville, MD: Agency for Healthcare Research and Quality. 2008. AHRQ Publication No. 08-0048. Available at: < <http://www.ahrq.gov/research/findings/final-reports/advisorycouncil/advisorycouncil.pdf>>. Accessed May 11, 2015.

2. Mackean G, and the Patient Experience Team. Involving Patient Experience Advisors on Region Committees and Teams. Calgary: Calgary Health Region; 2007.

3. Gillian Fletcher JB. Voices in action resource book. Salt Lake City: College of Health; 2001.

4. Health Canada. Health Canada Policy Toolkit for Public Involvement in Decision Making. Available at: <http://www.hc-sc.gc.ca/ahc-asc/pubs/_public-consult/2000decision/index-eng.php>. Accessed May 11, 2015.

5. Institute for Patient- and Family-Centered Care. Partnering with patients and families to design a patient- and family-centered health care system: Recommendations and promising practices. Available at: <http://www.ipfcc.org/pdf/PartneringwithPatientsandFamilies.pdf>. Accessed May 11, 2015.

6. Martin DK, Abelson J, Singer PA. Participation in health care priority-setting through the eyes of the participants. J Health Serv Res Policy. 2002;7:222-9.

7. National Health Service Modernisation Agency. Involving patients and carers. Improvement Leader's Guide. Available at: <<http://www.evidenceintopractice.scot.nhs.uk/media/135286/involving_patients_and_carers_guide.pdf>>. Accessed May 11, 2015.

8. Hall MF. Looking to improve financial results? Start by listening to patients. Healthc Financ Manage. 2008;62:76-80.

**Appendix Table A. Program Materials**

| **Booklet** | **Description** | **Audience** | **Contents** |
| --- | --- | --- | --- |
| Patient Engagement Toolkit | Easy to Use Step-by-Step “How To” Guide to Patient Engagement | Version for Coaches & Version for Team Members | Overview of Patient Engagement; 6 Step “How To” Worksheet; appendices of sample materials; references |
| Patient Partner Welcome Packet template | A customizable packet for teams to supply to patients | Patients | Introduction to patient role; Introduction to QI efforts; tips for successful service; confidentiality agreement |
| Patient Partner QI Packet: For the Truly “QI” Curious | Offers more detail about QI methods | Patients | Introduction to PDCA cycle, process mapping, and fishbone diagramming |
| Engaging Patient Partners for Better Care: Getting Started & Vital Resources | Internal guide to relevant policies and resources | Clinic Managers, Coaches, Team Members | HIPAA demystified, confidentiality concerns addressed, authorizations for publicity efforts |

QI = Quality Improvement; PDCA = Plan-Do-Check-Act; HIPAA = Health Insurance Portability and Accountability Act

**Appendix Table B. Six Steps of the “How To” Guide to Patient Engagement**

| **Step** | **Description** | **Tips** |
| --- | --- | --- |
| Discern | Surface and clarify team members’ opinions about the value patients add to quality improvement efforts | Understand key concerns of team members  Review the myths and facts |
| Match Engagement Methods with Projects | Decide on levels and methods of engaging patients best suited to the team’s quality improvement objective | Encourage any form of engagement early on  Develop a sound understanding of the levels and possible methods |
| Define the Job for Patients | Write a job description and clarify the role of patient(s) in the redesign effort | Include specific commitments (time and activities) patients must make  Use examples |
| Recruit Patients | Identify candidates and interview / have discussion with potential patients | Rely on knowledge of physicians and clinical staff for recommendations  Provide opportunity for patients to volunteer |
| Welcome Patients | Orient patients to the team and engage in welcoming behaviors | Address barriers to participation (e.g., transportation, child care, timing)  Use inclusive language and avoid use of jargon  Create participatory agendas; make necessary process changes to meetings including actively seeking input from all team members |
| Celebrate Successes and Capture Lessons | Measure and document outcomes of patient engagement  Recognize patients and teams | Communicate/ share value of patient engagement, including changes made as a result of patient engagement  Honor engaging teams and patient contributions publically |
